# IncI2 plasmid transfer and changes of intestinal microbiota in mice under β-lactam antibiotic pressure

**DOI:** 10.1186/s12917-025-04808-7

**Published:** 2025-05-15

**Authors:** Kaidi Liu, Junqi Liu, Yuting Su, Minge Wang, Tengfei Long, Liangxing Fang, Yufeng Zhou, Jian Sun, Xiaoping Liao

**Affiliations:** 1 National Risk Assessment Laboratory for Antimicrobial Resistance of Animal Original Bacteria, South China Agricultural University, Guangzhou, P. R. China; 2 School of Agricultural Science and Engineering, Liaocheng University, No.1 Hunan Road, Liaocheng, Shandong 252000 China; 3 Laboratory of Veterinary Pharmacology, College of Veterinary Medicine, South China Agricultural University, Guangzhou, 510642 P. R. China; 4 Guangdong Laboratory for Lingnan Modern Agriculture, Guangzhou, 510642 P. R. China

**Keywords:** Antibiotic resistance, IncI2 plasmid, β-lactam antibiotics, Conjugation transfer, Intestinal microbiota

## Abstract

**Background:**

β-lactam antibiotics represent the most widely utilized class of antimicrobial agents in livestock and poultry breeding. However, the effects of β-lactam antibiotics on conjugation transfer of IncI2 plasmids and the homeostasis of the mouse intestinal microbiota have not been thoroughly investigated.

**Results:**

The results revealed that the transfer of IncI2 plasmid was the highest for intra-specific *E. coli* and inter-specific transfer to *Salmonella* and *K. pneumoniae* occurred at much lower levels in the absence of β-lactam antibiotic selective pressure. Furthermore, inter-species and intra-species transfer of IncI2 plasmid was enhanced in the presence of sub-MIC levels of amoxicillin/clavulanate and cephalexin whereas ampicillin promoted only inter-species transfer. These results were consistent with in vivo observations where amoxicillin/clavulanate and cephalexin but not ampicillin promoted conjugation. Meanwhile, the intestinal microbiota was also disturbed following antibiotic treatment and *Proteobacteria* abundance increased while *Bacteroides* decreased. The gut microbiota could also be partially restored to initial levels after antibiotic cessation for 14 days.

**Conclusions:**

These findings highlight the potential risk of β-lactam antibiotics in promoting the spread of resistance plasmids and causing disruption to the intestinal microbiota.

**Supplementary Information:**

The online version contains supplementary material available at 10.1186/s12917-025-04808-7.

## Background

Antibiotics play an indispensable role in the prevention and treatment of infections caused by multidrug-resistant bacteria in livestock, poultry and companion animals [1, 2]. However, the routine use of antibiotics in intensive farming systems and unregulated use in veterinary clinics have accelerated antimicrobial resistance (AMR) emergence in animal pathogens, creating cross-species public health threats [3–5]. The dissemination of AMR among animal populations can significantly limit therapeutic options for veterinarians, potentially leading to treatment failure [6]. More notably, AMR poses serious threats to animal productivity and welfare, as it can exacerbate disease severity and increase mortality rates in both livestock and companion animals [7]. These clinical and economic consequences mainly are driven by the molecular mechanisms of resistance dissemination. Resistance phenotypes are usually a consequence of antibiotic resistance genes (ARGs) disseminated primarily through horizontal gene transfer (HGT) via conjugation, transformation and transduction [8]. Among these, conjugation serves as the predominant HGT mechanism for the dissemination of resistance plasmids, enabling their inter-species transmission among diverse bacterial species [8, 9].

Antibiotic overuse and misuse are the primary cause of the spread of resistance plasmids in bacterial populations [10]. Of particular concern in veterinary settings, subinhibitory antibiotic levels used in animal feed and veterinary medicine may induce transfer of plasmid-encoded resistance [11, 12]. For example, enrofloxacin and colistin can significantly increase the conjugation frequency of plasmids [9, 13]. Moreover, antibiotics also promoted plasmid transfer in vivo. Notably, in veterinary-relevant settings, the presence of tetracycline promoted the mobilization of RP4 plasmid and pRAS1 plasmid that lacked transfer functions in the mouse intestine [14, 15], highlighting a potential risk for antibiotic-driven resistance spread in animal hosts. In addition, the detection rate of resistance genes was relatively high in antibiotic-treated waterfowls and patients [16, 17]. These findings collectively underscore the role of veterinary antibiotic use in accelerating plasmid-mediated resistance transmission, thereby complicating disease control in animal populations. Beyond animal health, antibiotic use in livestock and companion animals poses environmental and public health risks [18]. Plasmid-borne resistance genes can be excreted in feces, contaminating soil and water, especially where animal waste is used as fertilizer or inadequately treated [19]. These ARGs may persist in environmental microbiota and transfer to human pathogens, highlighting the need for a One Health approach to address the broader impacts of veterinary antimicrobial use.

β-lactam antibiotics are extensively employed in animal breeding industry and veterinary clinics owing to their potent bactericidal activity, favorable safety profile, and high clinical efficacy [20]. Among these, ampicillin (AMP), amoxicillin (AMC) and cephalexin (CEP) are frequently employed in veterinary medicine for the empirical management of bacterial pyoderma in canines, the control of severe and systemic infections in swine herds, and therapeutic intervention of urinary tract infections in dogs [21–23]. Notably, the widespread use of β-lactams may exert indirect selection pressure on resistance mechanisms, including plasmid-mediated genes *mcr-1*, which encodes a phosphoethanolamine transferase conferring resistance to colistin-the last resort antibiotic used to treat multidrug-resistant Gram-negative infections. Our field surveillance in duck farms revealed a startling 32.4% prevalence of *mcr-1* in *E. coli* isolates from clinically healthy waterfowl, predominantly on conjugative IncI2 plasmids [24]. This asymptomatic spread in healthy ducks presents two key challenges for veterinary medicine: it undermines antimicrobial stewardship by maintaining resistance despite the ban on using colistin as a feed additive for growth promotion, and increases the risk of treatment failure when resistance plasmids transfer to pathogens responsible for common infections such as avian colibacillosis or swine enteritis. These findings suggest that veterinary use of β-lactam antibiotics may be driving the transmission of *mcr-1* through co-selection, even in the absence of direct colistin exposure. Therefore, the impact of β-lactams on the mobilization and horizontal transfer of *mcr-1*-carrying plasmids warrants further investigation.

The intestinal microbiota can also be dramatically altered with the use of antibiotics [25]. For example, previous studies have shown antibiotics are able to affect the composition of gut microbiota and destroy the homeostasis of intestinal microbiota, this is mainly due to the accumulation of unabsorbed antibiotics in colon and cecum following oral administration [26]. In addition, antibiotic pretreatment facilitated the gut colonization of *Salmonella* Typhimurium and *Listeria monocytogenes* indicating that antibiotics can also destroy intestinal barrier functions and increase colonization levels for exogenous pathogens [27, 28]. The recovery of gut microbiome following ciprofloxacin and vancomycin treatment has been thoroughly studied [29, 30], but these types of experiments have not been performed following β-lactam treatment.

In this study, we examined the effects of β-lactam antibiotics, particularly those commonly used in livestock production and companion animal medicine, on the transfer of *mcr-1*-positive IncI2 plasmids in vitro and in vivo and assessed their effects on the composition and recovery of the intestinal microbiota. These data were used to analyze potential relationships between antibiotic-induced dysbiosis and enhanced resistance gene dissemination. Our comprehensive analysis provides actionable insights for optimizing antimicrobial stewardship in livestock production and companion animal medicine, particularly regarding treatment protocols, withdrawal period determination, and probiotic intervention strategies to mitigate resistance spread.

## Material and methods

### Bacterial strains, plasmid and antibiotics

Conjugation experiments utilized the diaminopimelic acid (DAP) auxotroph *E. coli* 1917Δ*asd* and wild type *E. coli* SMS27 as donors, both carrying the IncI2 plasmid obtained from a wild-type *E. coli* isolate. The IncI2 plasmid is approximately 60 kb in size and carries the *mcr-1* gene, which encodes resistance to colistin. The *mcr-1* gene is flanked upstream by the insertion sequence *ISApl1* and downstream by *pap2*, forming the transposable element *ISApl1*-*mcr-1*-*pap2*. The recipient strains used for conjugation were *E. coli* C600 and wild-type *E. coli* strains, *Salmonella* ATCC14028 and wild-type *Salmonella* strains (including S. typhimurium and S. Indiana), *K. pneumoniae* ATCC700603 and wild-type *K. pneumoniae* strains, more detailed information about on wild-type recipient strains was listed in Additional file 1. Donors were cultured in Luria broth (LB) medium supplemented with 57 mg/L DAP. Ampicillin (AMP), amoxicillin/clavulanic acid (AMC) and cephalexin (CEP) were purchased from McLin Bio-Chemical Technology (Shanghai, China).

### Determination of MICs and growth curve

The minimal inhibitory concentrations (MIC) of antibiotics against donor and recipient strains were determined using the broth microdilution method and interpreted according to the CLSI standards [31]. *E. coli* ATCC 25922 was used as the quality control strain. All MIC experiments were conducted with biological triplicates.

Then, different sub-MICs of β-lactam antibiotics were added to cultures and growth curves were established following incubation at 37 °C. Specifically, overnight cultures of donor and recipient strains were diluted with fresh Luria–Bertani (LB)-broth to the optical density (OD_600_) of 0.1 ~ 0.15. Then the bacterial solution was diluted at a ratio of 1:100 and β-lactam antibiotics were added to each bacterium to attain a final concentration of 1/8 MIC, 1/4 MIC and 1/2 MIC. The bacterial culture was incubated at 37 °C and OD_600_ was measured every 1 h for 12 h. Each experiment was conducted with biological triplicates.

### Conjugation experiment

To assess the conjugative frequency of IncI2 plasmid, conjugation assays were performed using filter or broth mating method. Briefly, the donor and recipient bacteria were cultured at 37 °C and 180 rpm for about 4 h in LB broth medium. The bacterial cells were collected by centrifugation for 5 min at 6,000 × g and 4 °C, washed twice and was resuspended by PBS to achieve OD_600_ = 0.5. Then, the donor and recipient strains were mixed (1:1) and plated on LB agar plates or LB broth in the presence and absence of β-lactam antibiotics. After mating at 37 °C for 12 h, the mixture was diluted and plated on LB agar plates containing corresponding antibiotics to select transconjugants and recipients. Finally, PCR analysis of *mcr-1* gene carried by IncI2 plasmid was conducted to verify whether IncI2 plasmid is contained in transconjugants. The conjugative frequency was calculated according to the equation: Conjugative frequency = Number of transconjugants/Number of recipients [32]. All conjugative experiments were conducted in triplicate.

### Establishment of intestinal conjugative transfer model

The intestinal conjugation transfer model was constructed to evaluate the effect of β-lactam antibiotics on horizontal transfer of IncI2 plasmid in the mouse intestine as previously described [33]. Briefly, 5–6 weeks old female BALB/c mice (*n* = 6 per group) with body weights ranging from 18–22 g purchased from Guangdong Medical Laboratory Animal Center were used in the experiments. The sample size was determined based on previous studies involving the establishment of mouse models with bacteria [34–37]. Mice were randomly assigned to different experimental groups using a random number generator (GraphPad Prism 8.0) to ensure unbiased allocation and reduce potential selection bias. Each individual mouse was considered as one experimental unit in this model. The mice were housed in a temperature-controlled environment (22 ± 2 °C) with a relative humidity of 50–60% under a 12-h light/dark cycle. The mice were initially administered 5 g/L streptomycin orally for 3 days to disrupt the intestinal microbiota as previously described [38], followed by fresh water for 24 h to clear the streptomycin from the animal system. The mice were then given either AMP (150 mg/kg), AMC (50 mg/kg), CEP (50 mg/kg) or PBS (negative control) via gavage once a day for 3 days. The antibiotic doses used in our study were determined based on previous literature [39–42] and adjusted according to mouse body weight. Bacterial inoculums consisted of donor and recipient strains mixed 1:1 and were administered with a single dose gavage of 2 × 10^8^ colony-forming units CFU/mL for 200 μL. Fresh feces were collected from the animals after 24 h following inoculation and transconjugants and recipient strains were screened by plating on LB agar containing the appropriate antibiotics. It is important to note that as the personnel involved in animal handling need to be aware of the group assignments to ensure the correct implementation of the experimental protocol, blinding was not applied during the allocation and intervention stages. However, we employed single-blind methods during outcome assessment and data analysis to minimize bias as much as possible. In addition, animal welfare was monitored daily throughout the study. Specifically, mice were monitored daily for signs of severe weight loss, lethargy, reduced food/water intake, diarrhea, or abnormal behavior. If any of these conditions were met, the mouse was humanely euthanized. Notably, no mice were excluded due to ill health during the experimental period. All mice were humanely euthanized by CO₂ asphyxiation followed by cervical dislocation, in accordance with institutional animal care and use guidelines at the end of the experiment, and they were not used for any other purposes.

### 16S rRNA sequencing and analysis

The effects of β-lactam antibiotics on intestinal microbiota were further examined by treating mice as per above with either AMP (150 mg/kg), AMC (50 mg/kg) or CEP (50 mg/kg) via gavage for 3 days, each experimental group consisted of nine 5–6 weeks old female BALB/c mice (18–22 g), which purchased from Guangdong Medical Laboratory Animal Center. The sample size (*n* = 9 per group) was determined based on previous studies [43]. The approach used for assigning mice to different experimental groups, as well as the housing conditions, were consistent with those applied in the conjugative transfer model. Fresh feces were collected at baseline (Before antibiotic treatment, B0), treating for a day and three days (T1 and T3), and 1, 4, 6, 10 and 14 days after treatment (P1, P4, P6, P10, and P14, respectively). Notably, to reduce individual variability and ensure sufficient biomass for downstream genomic DNA extraction and sequencing analyses, we thoroughly mixed fecal samples from the three mice in each group to obtain one pooled sequencing sample according to the previous method [43], resulting in three sequencing samples per group. This method ensures reliable microbiota analysis while accounting for the variability in microbial composition. Therefore, each pooled fecal sample (consisting of feces from three mice per group) was considered as one experimental unit in the 16S rRNA sequencing experiments. Specifically, we combined precisely measured fecal aliquots (33 ± 1 mg wet weight) from three randomly selected mice per group using sterile techniques. Pooled samples were homogenized through vortex mixing in anaerobic conditions (3 min), homogenized using bead-beating (6.5 m/s, 45 s) in DNA Shield buffer, and centrifugation (500 × g, 2 min) to remove particulates, ensuring consistent sample processing. The samples were stored at −80 °C until extraction. Microbial genomic DNA was extracted from approximately 100 mg fecal specimens using the QIAamp Power Fecal DNA isolation kit (Cat No 12830–50; Qiagen, Hilden, Germany) according to the manufacturers’ instructions. The quality and concentration of DNA were assessed using a NanoDrop2000 spectrophotometer (Thermo Fisher Scientific, Waltham, MA, USA). The V3-V4 regions of the 16S rRNA were amplified using specific primers: 341 F (5′-CCTAYGGGRBGCASCAG-3′) and 806R (5′-GGACTACNNGGGTATCTAAT-3′). PCR amplification was performed under the following thermal cycling conditions: initial denaturation at 98 °C for 1 min; 30 cycles of denaturation at 98 °C for 10 s, primer annealing at 50 °C for 30 s, and extension at 72 °C for 30 s; followed by a final extension at 72 °C for 5 min. The purified PCR amplicons were sequenced on an Illumina Nova6000 platform with the PE250 mode in Novogene Bioinformatics Technology (Beijing, China). Moreover, it should be emphasized that the blinding methods and animal welfare procedures used in this experiment were consistent with the intestinal conjugative transfer model described above.

All raw sequencing data were processed using QIIME2 (v2023.5). After quality filtering, reads were denoised via DADA2 to obtain amplicon sequence variants (ASVs). Default parameters were applied for all steps [44]. Quality-filtered sequences were clustered into operational taxonomic units (OTUs) at a 97% similarity threshold using the UPARSE pipeline (Uparse v7.0.1001, http://www.drive5.com/uparse/). Subsequently, taxonomic annotation was performed using the SSU rRNA database (SILVA 138.1, http://www.arb-silva.de/) with a similarity threshold of 0.8–1.0, generating classification information that was statistically analyzed at both phylum and genus levels. Multiple sequence alignment of all OTU representative sequences was performed using MUSCLE software (v3.8.31, http://www.drive5.com/muscle/). Alpha diversity indices were calculated using QIIME2 (v2023.5) and subsequently visualized through the R project (v3.4.1). A principal component analysis (PCA) with a permutation multivariate analysis of variance (Adonis) was applied to describe beta diversity of predicted functions.

### Statistical analysis and data visualization

Prism 8.0 (Graphpad, Boston, MA, USA), SPSS 25.0 (SPSS, Chicago, USA) and R Studio (version 3.4.1) were used for data analysis and visualization. The results were analyzed by analysis of variance (ANOVA) and independent-sample t test, with the Benjamini–Hochberg correction. The corrected *P* values of < 0.05 were considered to indicate statistical significance.

## Results

### Analysis of inter- and intra-species plasmid transfer

**Fig. 1:**
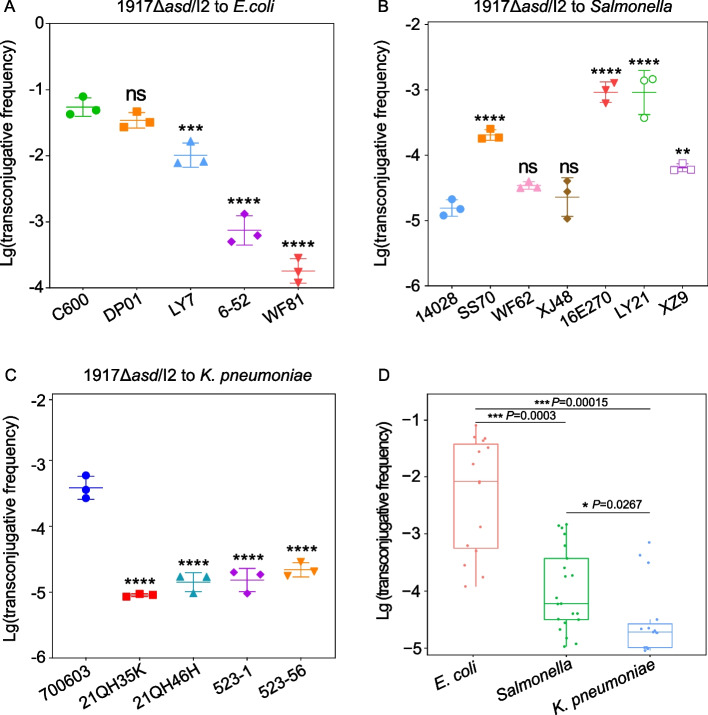
Inter- and intra-specific conjugative frequency of IncI2 plasmid in the absence of antibiotic selective pressure. Conjugative frequency for *E. coli* donor and (**A**) *E. coli* recipient (**B**) *Salmonella* recipient and (**C**) *K. pneumoniae* recipient (**D**) Comparison of conjugative frequency of IncI2 plasmid. (ns *P* > 0.05; * *P* < 0.05; ** *P* < 0.01; *** *P* < 0.001; **** *P* < 0.0001)

The initial step in these experiments was to evaluate the transferability of IncI2 plasmid from *E. coli* 1917Δ*asd* to *E. coli* C600, *Salmonella* 14,028, *K. pneumoniae* 700,603, and wild-type strains. The results showed that all three Enterobacterial species were able to act as recipients of the IncI2 plasmid and the MIC of colistin is the same for both the transconjugants and donor bacteria. The frequency of transfer varied with species as follows: *E. coli* 5 × 10^–4^ to 7.88 × 10^–2^ (Fig. 1A), *Salmonella* 1.05 × 10^–5^ to 1.46 × 10^–3^ (Fig. 1B) and *K. pneumoniae* 9.08 × 10^–6^ to 7.07 × 10^–4^ (Fig. 1C). The conjugative frequency of IncI2 plasmids was higher to most wild-type *Salmonella* strains than to the standard strain, whereas transfer to *E. coli* and *K. pneumoniae* was more efficient in standard strain than in wild-type isolates (*P* < 0.05). Moreover, the comparative analysis of the conjugative frequency of IncI2 plasmid indicated that the transferability of IncI2 plasmid to *E. coli* was significantly higher than *Salmonella* and *K. pneumoniae* (*P* < 0.05) (Fig. 1D).

### Conjugative frequency of plasmid was enhanced by β-lactam antibiotics

The effect of β-lactam antibiotics (AMP, AMC, and CEP) on the conjugation frequency of IncI2 plasmid was further evaluated using donor strain *E. coli* 1917Δ*asd*. We first established the MICs for the donor and recipient strains (Additional file 2). Subsequently, bacterial growth was monitored under sub-inhibitory concentrations of AMP, AMC and CEP. Growth of these strains was not significantly affected by 1/8 MIC of antibiotics (Additional file 3, Additional file 4). Therefore, 1/8 MIC of β-lactam antibiotics was selected to assess the effect on conjugation frequency.

**Fig. 2:**
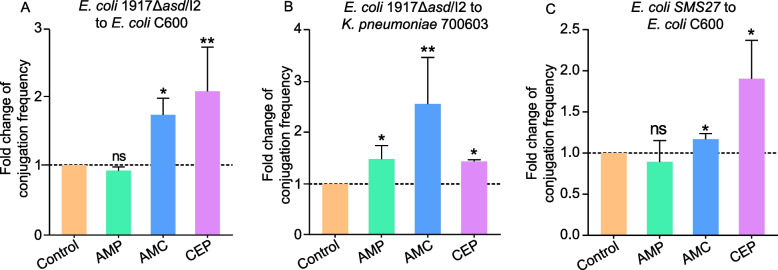
Effects of sub-MIC levels of β-lactam antibiotics on the conjugation frequency of IncI2 plasmid. Fold change of IncI2 plasmid transfer for (**A**) intra-species and (**B**) inter-species mating. **C** Fold change of conjugation frequency of IncI2 plasmid between wild type *E. coli* SMS27 and E. coli C600. (ns *P* > 0.05; * *P* < 0.05; ** *P* < 0.01)

As shown in Fig. 2, the presence of sub-MIC β-lactams antibiotic significantly promoted both inter- and intra-species conjugative transfer. Particularly, AMC and CEP significantly enhanced intra-species transfer by 1.75- and 2.05-fold (*P* < 0.05) (Fig. 2A). Altogether, inter-species conjugation frequency increased by 1.49-, 2.59-, and 1.45-fold in the presence of AMP, AMC, and CEP (*P* < 0.05) (Fig. 2B). We further utilized a wild-type *E. coli* strain SMS27 as donor to explore whether the effects of antibiotics on plasmid were related to the donors. Interestingly, conjugation frequency of IncI2 plasmid from *E. coli* SMS27 to C600 was increased by 1.13- and 1.90-fold under the pressure of AMC and CEP, which is consistent with the result that *E. coli* 1917Δ*asd*/I2 strain served as donor (*P* < 0.05) (Fig. 2C). Taken together, these experiments demonstrated that AMC and CEP significantly promoted both inter-species and intra-species conjugative transfer of IncI2 plasmid while AMP only affected inter-species transfer.

### β-lactam antibiotics promoted plasmid transfer in the mouse intestine

**Fig. 3:**
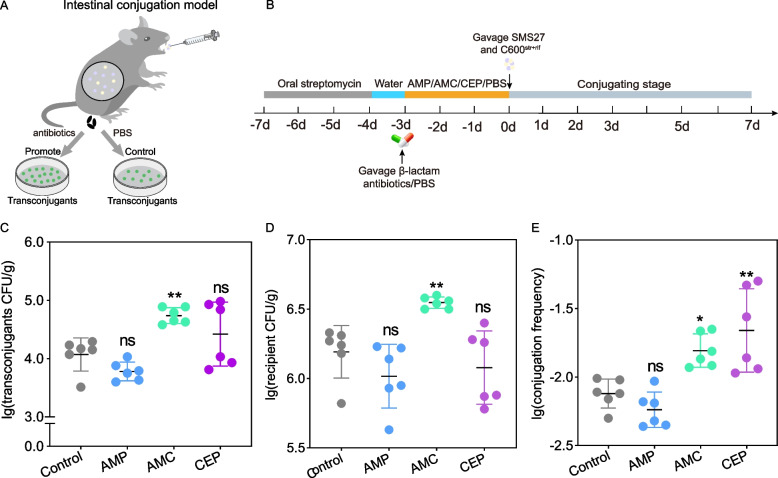
Effect of β-lactam antibiotics on the horizontal transfer of IncI2 plasmid in vivo over 24 h. **A** Establishment of mouse intestinal conjugation transfer model. **B** Flow chart of mouse intestinal conjugation transfer experiment. **C** Numbers of transconjugants. **D** Numbers of recipient bacteria. **E** Conjugation frequency of IncI2 plasmid in the mouse intestinal tract. (ns *P* > 0.05; * *P* < 0.05; ** *P* < 0.01)

A mouse intestinal model of plasmid transfer was established to test the effects of AMP, AMC, and CEP on plasmid transfer in vivo (Fig. 3A, B, Additional file 5). The numbers of transconjugants and recipients were evaluated 24 h following antibiotic treatment and found that the number of transconjugants and recipients in AMC-treated group were significantly higher than controls (*P* < 0.05). In addition, most of the transconjugants in CEP-treated group were also higher than control group but none were observed in the recipients (*P* < 0.05) (Fig. 3C and D). The AMC and CEP treatment groups displayed increases in plasmid transfer of 2.08- (1.61 × 10^–2^) and 3.45-fold (2.67 × 10^–2^) compared with controls (7.74 × 10^–3^) in the mouse intestine (*P* < 0.05) (Fig. 3E). Collectively, these results demonstrate that β-lactam antibiotics AMC and CEP promoted the conjugative transfer of IncI2 plasmid in mouse intestine.

### β-lactam antibiotics altered the diversity and composition of gut microbiota

**Fig. 4:**
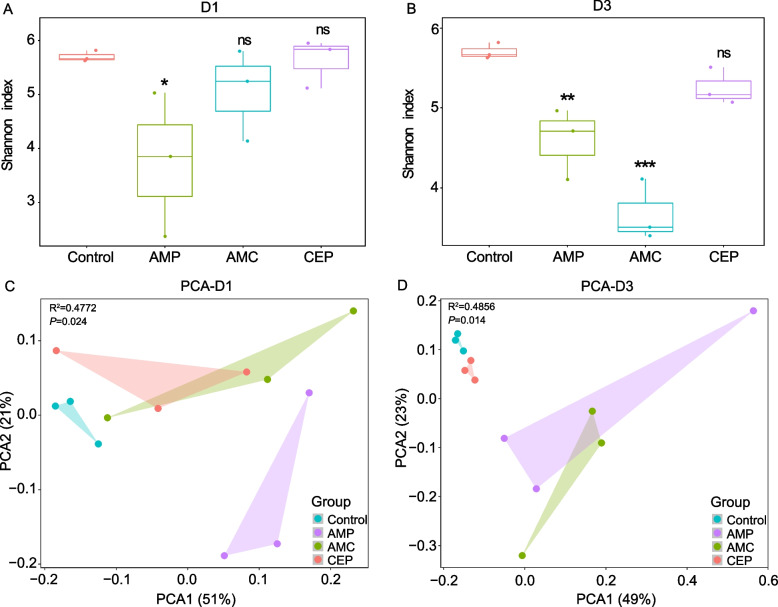
Change of richness and diversity in gut microbiota of mice. Microbiome richness after antibiotic treatment for (**A**) 1 day and (**B**) 3 days. PCA analysis following treatment for (**C**) 1 day and (**D**) 3 days. (ns *P* > 0.05; * *P* < 0.05; ** *P* < 0.01;*** *P* < 0.001)

The effects of β-lactam antibiotic on the intestinal microbiota were investigated by 16S rRNA sequencing in our experimental mice. Notably, the mice in this study did not receive donor and recipient bacteria. Therefore, exogenous bacteria did not directly interfere with the microbiota. The results demonstrated that only AMP significantly altered the richness and evenness of the mouse microbiota with a 1-day treatment (*P* < 0.05) (Fig. 4A). As the treatment duration extended to 3 days, both AMP and AMC significantly decreased the richness and evenness of the microbiomes (*P* < 0.05) (Fig. 4B). The results indicated the alteration of richness and evenness in the intestinal microbiota became more pronounced with the prolongation of treatment time. Additionally, the components PCA1 and PCA2 of the Bray–Curtis distances each explained 72% of the variance. The samples in the treatment group were also significantly separated from the control group samples (Fig. 4C and D). Adonis tests (analysis of variance using distance matrices) also indicated that the differences in gut microbiota composition caused by the treatment with β-lactams at one day (R^2^ = 0.4772, *P* < 0.05) and three days (R^2^ = 0.4856, *P* < 0.05) significantly differed. These results indicated that β-lactams antibiotic exposure reduced the microbiome richness and remodeled the gut microbiome.

**Fig. 5:**
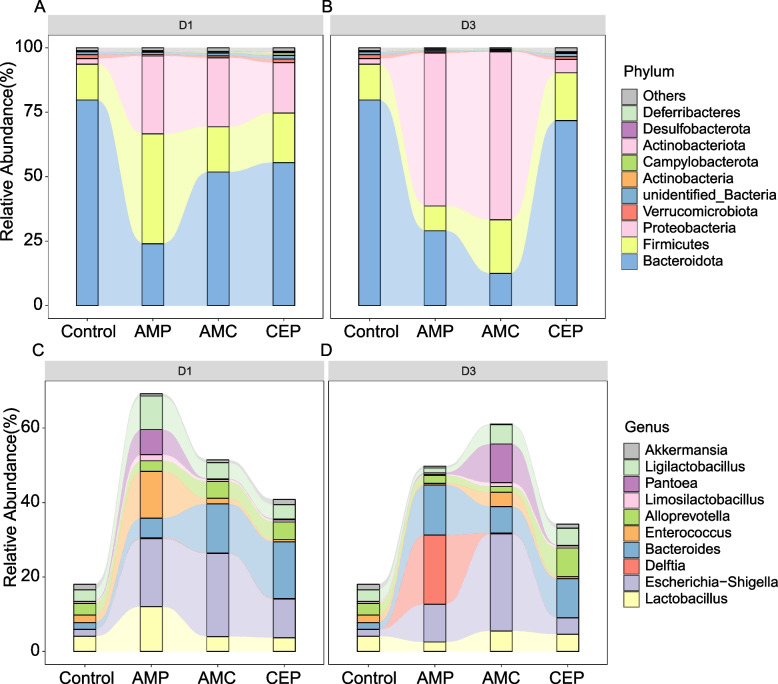
Alterations in the mouse gut microbiome following β-lactam treatments. Changes at the level of the (**A**, **B**) phylum and (**C**, **D**) genus

Next, the abundance and structure of gut microbiota at phylum and genus levels was explored. It was shown that *Bacteroidetes*, *Firmicutes* and *Proteobacteria* were the predominant taxonomic phyla in gut microbiota before and after antibiotic treatment. The abundance of *Proteobacteria* dramatically increased after treatment with AMP, AMC and CEP for one day and three days while the abundance of *Bacteroidetes* significantly decreased (*P* < 0.05) (Fig. 5A and B). Moreover, the abundance of *Escherichia-Shigella* and *E. coli* in the AMP, AMC, and CEP treatment groups were significantly higher than controls (*P* < 0.05) (Fig. 5C and D, Additional file 6). Notably, the increase of *Escherichia-Shigella* and *Escherichia coli* was the primary contributor to the high level of *Proteobacteria* abundance.

### Recovery of gut microbiota after β-lactam antibiotics withdrawal

**Fig. 6:**
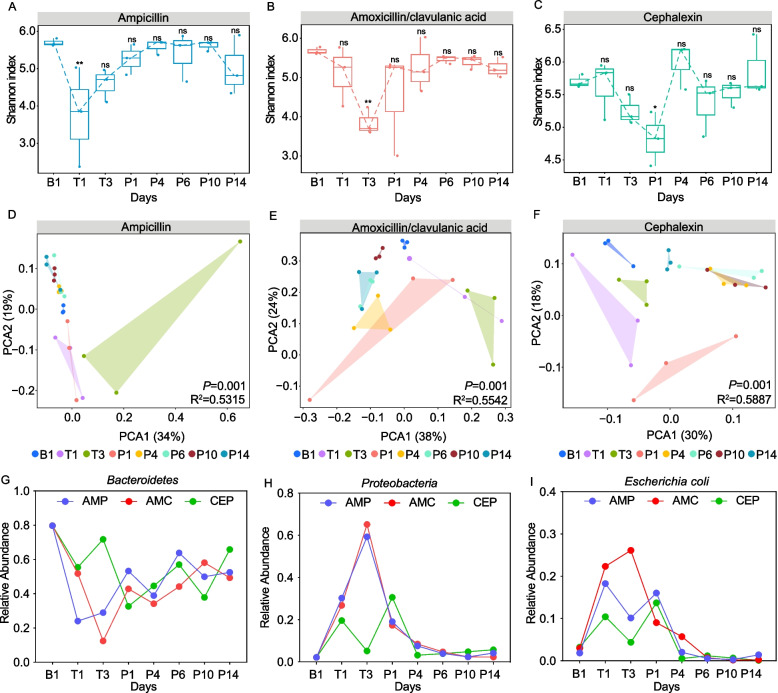
Recovery of gut microbiota following antibiotic withdrawal. **A**-**C** Richness. **D**-**F** PCA analysis. Relative abundance of (**G**) *Bacteroidetes* (**H**) *Proteobacteria* and (**I**) *E. coli*. (ns *P* > 0.05; * *P* < 0.05; ** *P* < 0.01)

The recovery of intestinal microbiota after β-lactam antibiotics treatment and withdrawal was further characterized. The gut microbiota was monitored for 14 successive days and it was found that microbiome richness was lowest at T1 in AMP-treated group and T3 in AMC-treated group (*P* < 0.05) and recovered to the original level after 14 days of antibiotic cessation (Fig. 6A and B). In contrast, community richness reached its lowest level at P1 and began to increase at P4 for the CEP-treated group (Fig. 6C). PCA analysis demonstrated the gut microbial composition at P1, P4, P6, P10 and P14 clustered more closely with the composition at B0 rather than the T1 and T3 in the AMP and AMC groups (Fig. 6D and E). This indicated that the differences gradually reduced from P1 between treated and control groups after the withdrawal of AMP and AMC. However, in the CEP-treated group, the differences in gut microbial composition were even greater at P1 and only began to recover at P4, which is consistent with the richness (Fig. 6F). In summary, alterations in the community structure of the mouse gut microbiota were partially restored 14 days after the withdrawal of all three β-lactam antibiotics.

Subsequently, the recovery of *Bacteroidetes*, *Proteobacteria*, and *Escherichia coli*, whose abundances changed significantly during the treatment, were further investigated. *Bacteroidetes* abundance decreased in a time-dependent manner with β-lactam antibiotic treatment but gradually recovered after the antibiotics were discontinued and recovered after the antibiotics were discontinued (Fig. 6G). In contrast, *Proteobacteria* and *E. coli* levels increased during antibiotic treatment and dropped in a time-dependent manner following withdrawal (Fig. 6H and I). In summary, these data linked the baseline level restoration of intestinal microbiota following antimicrobial withdrawal to alterations in these dominant bacterial community members.

## Discussion

Plasmids play an important role in the transmission of antibiotic resistance genes. IncI2, IncX4, and IncHI2 plasmids were considered as the important transmission vectors of *mcr*-*1* [45]. IncI2 plasmids are also the most frequently reported to possess *mcr-1* in colistin-resistant Enterobacteriaceae. Therefore, it is necessary to study the transmission of IncI2 plasmid among Enterobacteriaceae [46]. We found that conjugation frequency of IncI2 plasmid from *E. coli* donors to *E. coli*, *Salmonella* and *K. pneumoniae* were 5 × 10^–4^ to 7.88 × 10^–2^, 1.05 × 10^–5^ to 1.46 × 10^–3^, and 9.08 × 10^–6^ to 7.07 × 10^–4^ in the absence of antibiotic pressure. These results were similar to a previous study that *mcr-1* could transfer among *E. coli* through IncI2 plasmid obtained from tertiary hospitals at a conjugation frequency of 10^–3^ ~ 10^–1^ [47] and higher than those of another study, which reported a conjugation frequency of IncI2 plasmid at 1 × 10^–7^ [48]. These differences may be due to different conjugation time, temperature and conjugation system. Moreover, IncI2 plasmid had the highest conjugation frequency to *E. coli*, followed by *Salmonella* and *K. pneumoniae*, suggesting that conjugation frequency of IncI2 plasmid may also depend on the recipient bacteria. Consistent with our results, a recent study also revealed that conjugation frequency of RP4 plasmid for intra -species transfer (3 × 10^–4^) was much higher than inter-species (1 × 10^–6^) [49]. These studies collectively reflect an underlying issue that is plasmid transfer occurred more frequently between mating pairs of the same species than between those of different species, leading to a higher conjugation frequency within the same species compared to between different species [50, 51]. Therefore, our results demonstrated that IncI2 plasmid had a higher transferability to *E. coli* than *salmonella* and *K. pneumoniae*, which confirms the previous conclusion that *E. coli* (91%) is the most popular strain carrying *mcr-1*, followed by *Salmonella* (7%) and *K. pneumoniae* (2%) [52].

Antibiotic pressure plays a crucial role in promoting the transfer of resistant plasmids [53]. β-lactam antibiotics are one of the most successful chemical classes of antibiotics and are widely used in the veterinary clinic to treat infections [20]. In this study, sub-MICs (1/8 × MIC) of AMC and CEP significantly enhanced inter- and intra-specific transfer while AMP promoted only inter-species transfer. Similarly, the conjugative frequency of IncI1 plasmid was increased by about 2.5-fold under the pressure of sub-MIC cefotaxime and ampicillin [54]. Nevertheless, the promotion effect of colistin at bactericidal concentration on IncI2 plasmid was significantly higher than our results [13], a possible explanation lies in differences in the type or concentration of antibiotics. Antibiotics have also been shown to promote plasmid transfer in the animal gut environment [54]. Therefore, the association of antibiotics with increased conjugation frequency of plasmids in the gut have also been confirmed. The current study found that AMC and CEP promoted HGT of IncI2 plasmid in mouse gut while AMP had no significant effect, which is consistent with the results in vitro. A previous study had also reported that ceftriaxone accelerated the emergence of AmpC β-lactamase-producing Enterobacter in the intestinal microbiota of hospitalized patients [55]. In addition, ceftiofur or enrofloxacin treatments have been associated with the spread of *bla*_CTX-M_-positive plasmids in the mouse gut [56]. In contrast, ampicillin has been confirmed to promote the transfer of *bla*_NDM_-positive plasmid in the mouse gut [57]. Collectively, these results provide sufficient evidence that β-lactam antibiotics can promote the spread of plasmid-mediated resistance genes.

The presence of β-lactam antibiotics also promoted the spread of colistin resistance and produced pronounced alterations in the mouse gut microbiome. When mice were treated only with β-lactam antibiotics without interference from donor and recipient bacteria, the richness of gut microbiota decreased and the composition was significantly altered in this study. β-lactam antibiotics additionally led to decreased *Bacteroides* and increased *Proteobacteria* abundance. These results are similar to previous studies that exposure to meropenem, cefoperazone/sulbactam and aztreonam significantly decreased the diversity of intestinal microbiota and short-term cefoperazone/sulbactam treatment reduced *Bacteroides* abundance [58]. *Escherichia-Shigella* and *E. coli* levels significantly increased in our study following β-lactam antibiotics treatment suggesting *Proteobacteria* increases were primarily due to increases in *Escherichia-Shigella* and *E. coli*. Interestingly, colonization of exogenous pathogens could be inhibited by the colonization barrier forming abilities of intestinal microbiota [26]. *Bacteroides* is a successful competitor in the intestinal ecological environment and can prevent the colonization of exogenous bacteria in the intestine [59]. In our study, short-term treatment with AMP, AMC and CEP reduced Bacteroides abundance and this may have led to decreased colonization resistance thereby facilitating the colonization and effective contact between donor and recipient bacteria in the intestine to further affect plasmid transfer. However, the colonization level of recipient bacteria was only significantly increased in the AMC group compared with controls and conjugation frequency of IncI2 plasmid was also increased. We found no significant changes for the other two antibiotic groups although conjugation frequency was increased in the CEP group. In addition to the colonization of donor and recipient bacteria, other possible factors to promote the horizontal transfer of plasmids in the intestinal tract still requires further exploration.

Despite the severe disruption of intestinal microbiota structure by β-lactam antibiotics in our experiments, partial recovery of diversity and composition of gut microbiota were observed 14 days after antibiotic cessation. A previous study indicated that the gut microbiota was able to recover to near-baseline composition following vancomycin withdrawal [30]. Moreover, similar result was also noted when ciprofloxacin treatments were halted in humans where gut microbiomes began to return to their initial states after one or two weeks following antibiotic discontinuation [28, 30]. In particular, the richness of the microbiota almost recovered to its original state within 14 days. In contrast, the composition of the intestinal microbiota remained altered until the end of the experiment, this may be due to that the gut microbiome was severely disrupted and took longer to recover [60]. In addition, the relative abundance of *Proteobacteria* and E. coli returned to initial level after 14 days following antibiotics cessation while *Bacteroidetes* abundance was still significantly lower than pre-treatment levels, this indicated that *Bacteroidetes* in the intestinal microbiota probably have been permanently lost or severely depleted due to the treatment [60].

There are still several limitations in our study. Firstly, we did not consider the effect of β-lactam antibiotics on the transfer of other plasmids carrying *mcr-1* or plasmids carrying other resistance genes (such as *bla*_NDM_ or *tet*(X)). Secondly, the practice of pooling fecal samples from every three mice prior to 16S rRNA sequencing limited the statistical analysis of individual samples within each group. Thirdly, this study lacked other antibiotics, especially the effect of colistin on the spread of the plasmid IncI2. Fourthly, the antibiotic treatment dose in this study was relatively limited in vivo. Therefore, the relationship between the plasmid-promoting effect of antibiotics and therapeutic antibiotic doses requires further investigation. Finally, the specific relationship between the gut microbiota and the transfer of resistant plasmids remains unclear.

## Conclusion

This study provides critical insights for veterinary medicine regarding antimicrobial resistance (AMR) development and the imperative for judicious antibiotic use. It reveals that the IncI2 plasmid, carrying the *mcr-1* gene, transfers most frequently between *E. coli*, followed by *Salmonella* and *K. pneumoniae*, even without antibiotic pressure. Notably, β-lactam antibiotics, like AMP, AMC, and CEP, further promote this plasmid transfer both in vitro and in the mouse gut, accelerating the spread of resistance. Additionally, these antibiotics disrupt gut microbiota diversity and composition, with some bacterial groups, such as *Bacteroides*, failing to recover post-treatment. These findings underscore that β-lactam antibiotics not only exacerbate resistance gene dissemination but also cause long-term ecological imbalances in the gut microbiome. These findings underscore the urgent need for optimized antimicrobial stewardship in both livestock production and companion animal medicine, warranting more stringent regulations on β-lactam usage in animal breeding industry and veterinary clinical practice.

## Supplementary Information

Additional file 1.

Additional file 2.

Additional file 3.

Additional file 4.

Additional file 5.

Additional file 6.

Additional file 7.

## Data Availability

All data generated or analyzed during this study are included in this published article and its supplementary information files. The datasets generated during the current study are available in the NCBI database [accession number: PRJNA1081687. https://www.ncbi.nlm.nih.gov/bioproject/PRJNA1081687].
