# Supplementary material for: IncI2 plasmid transfer and changes of intestinal microbiota in mice under β-lactam antibiotic pressure
Source: BMC Vet Res. 2025 May 15;21:343. doi: 10.1186/s12917-025-04808-7 (PMC12080001; doi:10.1186/s12917-025-04808-7)
Supplement: Supplementary file 1 — Additional file 1. [file 12917_2025_4808_MOESM1_ESM.pptx]

## Slide 1
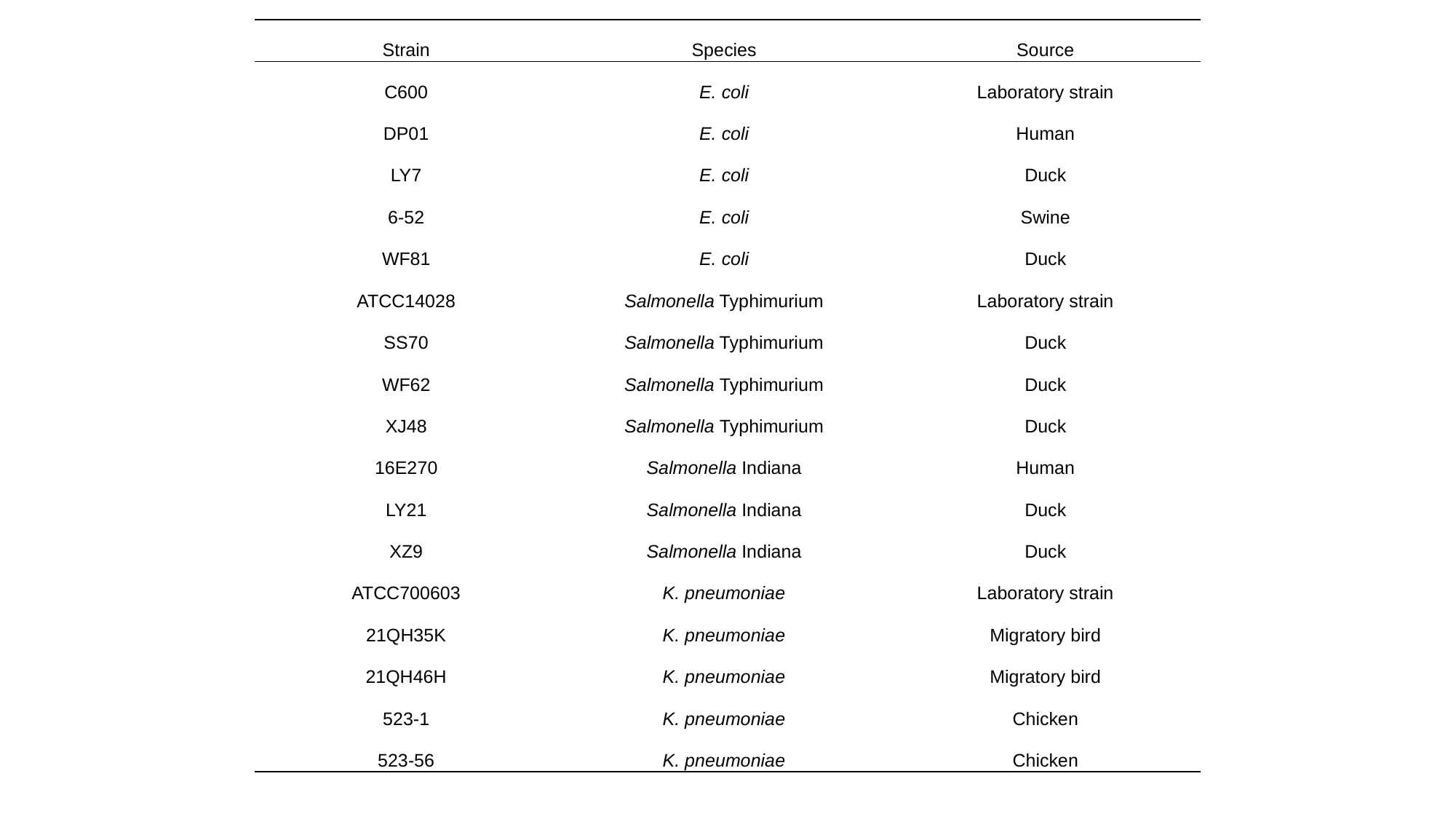

| Strain | Species | Source |
| --- | --- | --- |
| C600 | E. coli | Laboratory strain |
| DP01 | E. coli | Human |
| LY7 | E. coli | Duck |
| 6-52 | E. coli | Swine |
| WF81 | E. coli | Duck |
| ATCC14028 | Salmonella Typhimurium | Laboratory strain |
| SS70 | Salmonella Typhimurium | Duck |
| WF62 | Salmonella Typhimurium | Duck |
| XJ48 | Salmonella Typhimurium | Duck |
| 16E270 | Salmonella Indiana | Human |
| LY21 | Salmonella Indiana | Duck |
| XZ9 | Salmonella Indiana | Duck |
| ATCC700603 | K. pneumoniae | Laboratory strain |
| 21QH35K | K. pneumoniae | Migratory bird |
| 21QH46H | K. pneumoniae | Migratory bird |
| 523-1 | K. pneumoniae | Chicken |
| 523-56 | K. pneumoniae | Chicken |
