# Supplementary material for: IncI2 plasmid transfer and changes of intestinal microbiota in mice under β-lactam antibiotic pressure
Source: BMC Vet Res. 2025 May 15;21:343. doi: 10.1186/s12917-025-04808-7 (PMC12080001; doi:10.1186/s12917-025-04808-7)
Supplement: Supplementary file 2 — Additional file 2. [file 12917_2025_4808_MOESM2_ESM.pptx]

## Slide 1
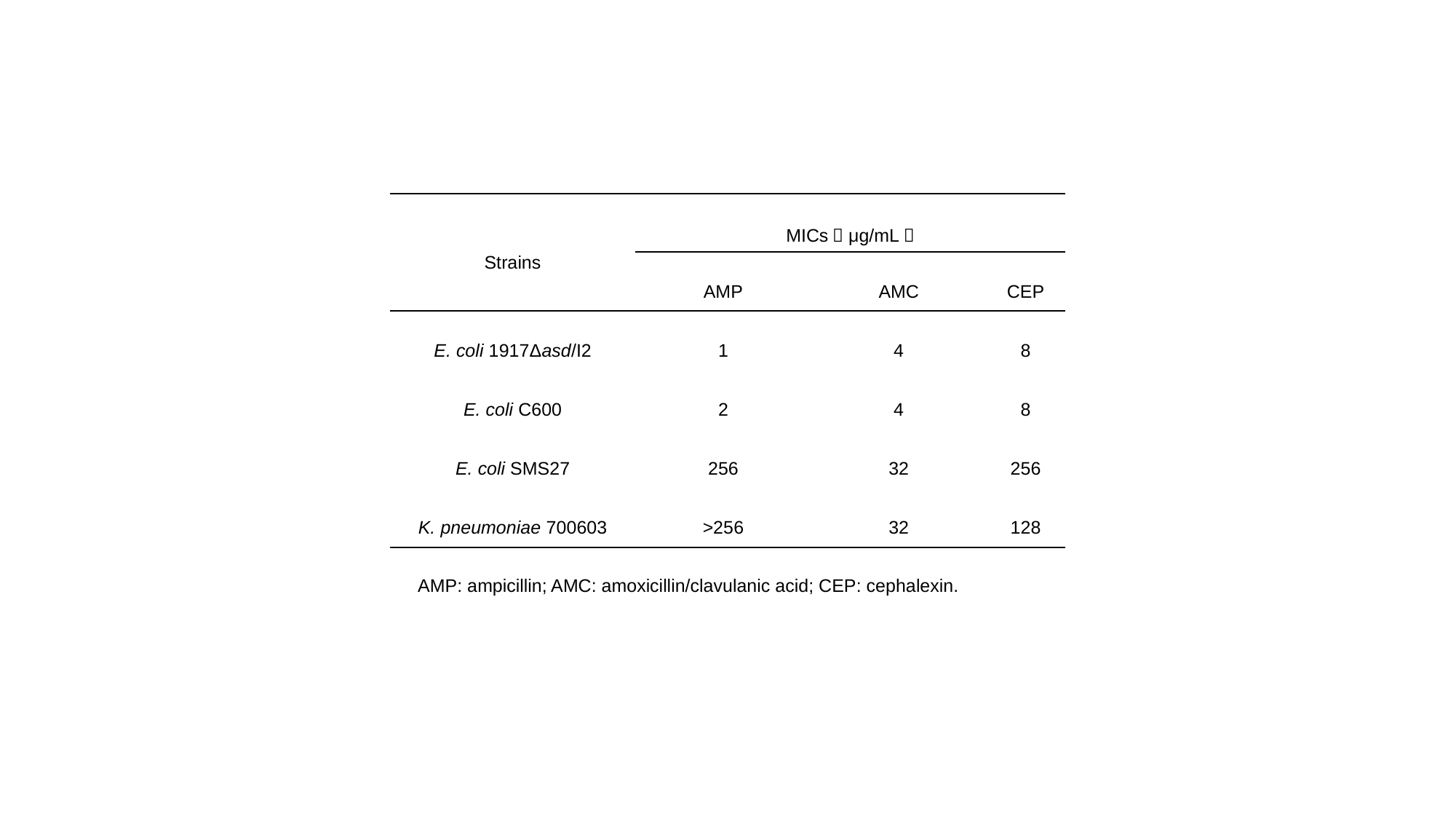

| Strains | MICs（μg/mL） | | |
| --- | --- | --- | --- |
| | AMP | AMC | CEP |
| E. coli 1917Δasd/I2 | 1 | 4 | 8 |
| E. coli C600 | 2 | 4 | 8 |
| E. coli SMS27 | 256 | 32 | 256 |
| K. pneumoniae 700603 | >256 | 32 | 128 |
AMP: ampicillin; AMC: amoxicillin/clavulanic acid; CEP: cephalexin.
