# Supplementary material for: IncI2 plasmid transfer and changes of intestinal microbiota in mice under β-lactam antibiotic pressure
Source: BMC Vet Res. 2025 May 15;21:343. doi: 10.1186/s12917-025-04808-7 (PMC12080001; doi:10.1186/s12917-025-04808-7)
Supplement: Supplementary file 3 — Additional file 3. [file 12917_2025_4808_MOESM3_ESM.pptx]

## Slide 1
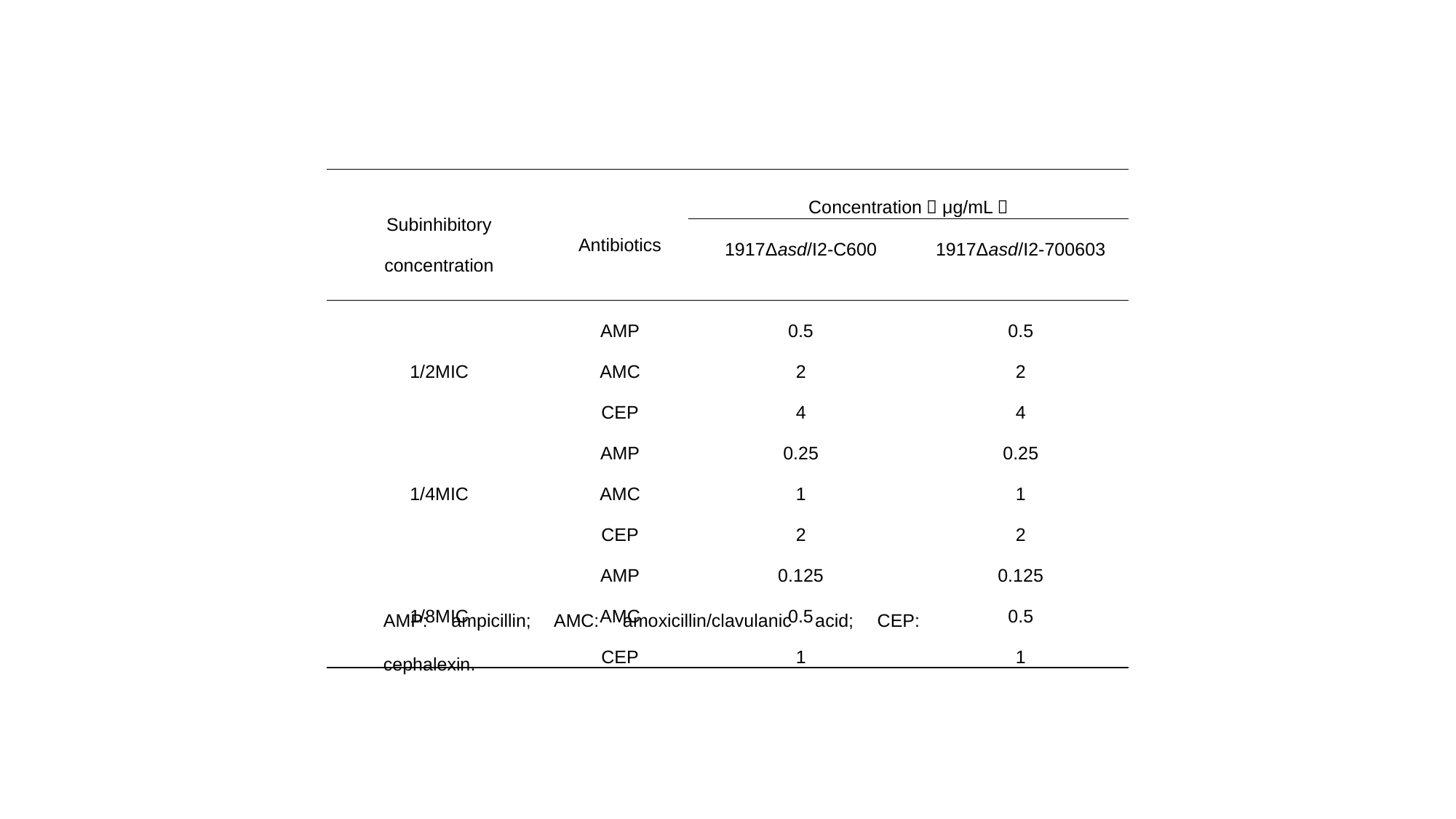

| Subinhibitory concentration | Antibiotics | Concentration（μg/mL） | |
| --- | --- | --- | --- |
| | | 1917Δasd/I2-C600 | 1917Δasd/I2-700603 |
| | AMP | 0.5 | 0.5 |
| 1/2MIC | AMC | 2 | 2 |
| | CEP | 4 | 4 |
| | AMP | 0.25 | 0.25 |
| 1/4MIC | AMC | 1 | 1 |
| | CEP | 2 | 2 |
| | AMP | 0.125 | 0.125 |
| 1/8MIC | AMC | 0.5 | 0.5 |
| | CEP | 1 | 1 |
AMP: ampicillin; AMC: amoxicillin/clavulanic acid; CEP: cephalexin.
