# Supplementary figures and images for: IncI2 plasmid transfer and changes of intestinal microbiota in mice under β-lactam antibiotic pressure
Source: BMC Vet Res. 2025 May 15;21:343. doi: 10.1186/s12917-025-04808-7 (PMC12080001; doi:10.1186/s12917-025-04808-7)

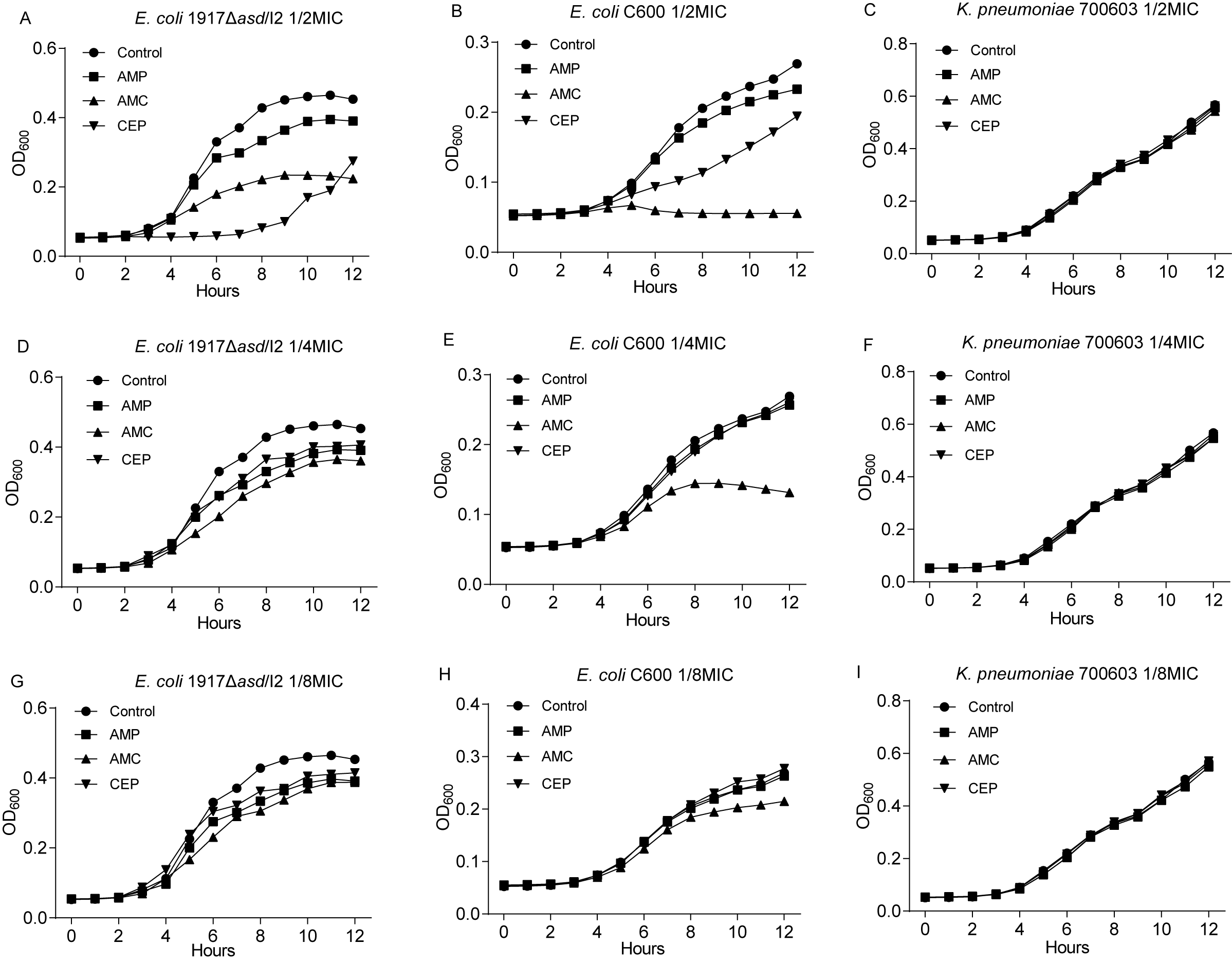

Supplement: Supplementary file 4 — Additional file 4. [file 12917_2025_4808_MOESM4_ESM.tif]

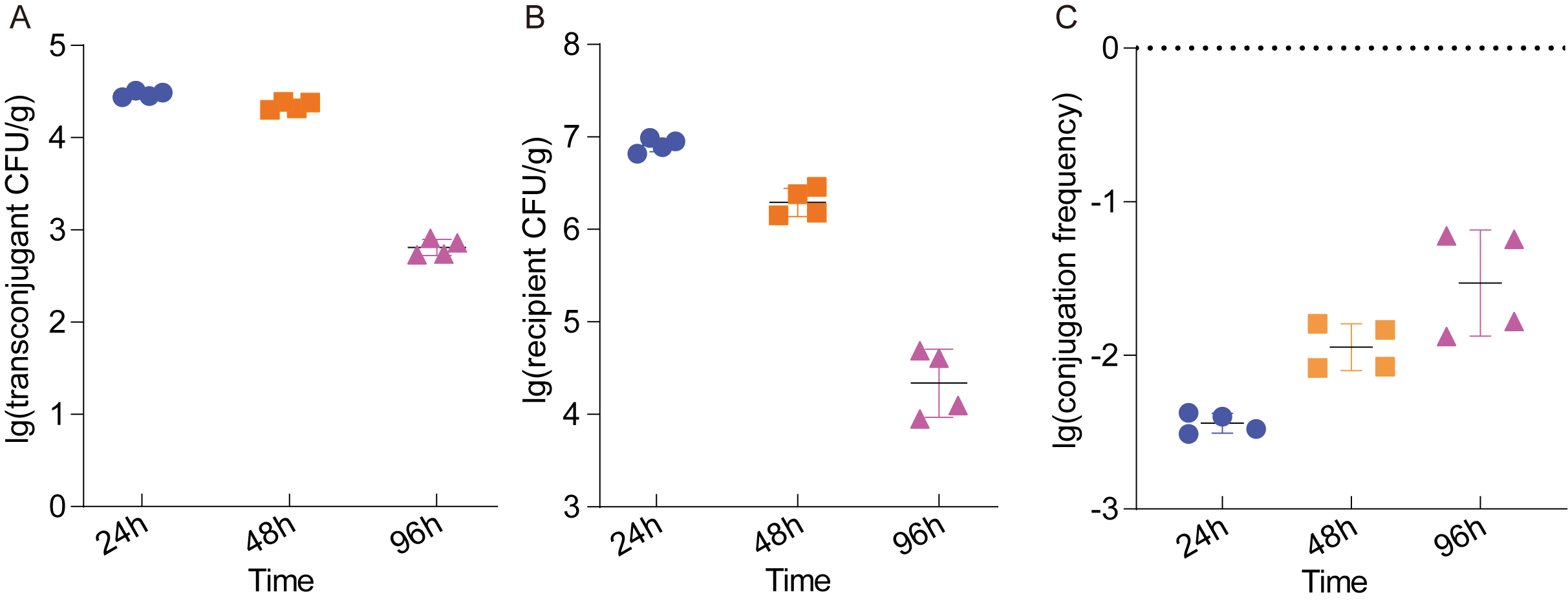

Supplement: Supplementary file 5 — Additional file 5. [file 12917_2025_4808_MOESM5_ESM.tif]

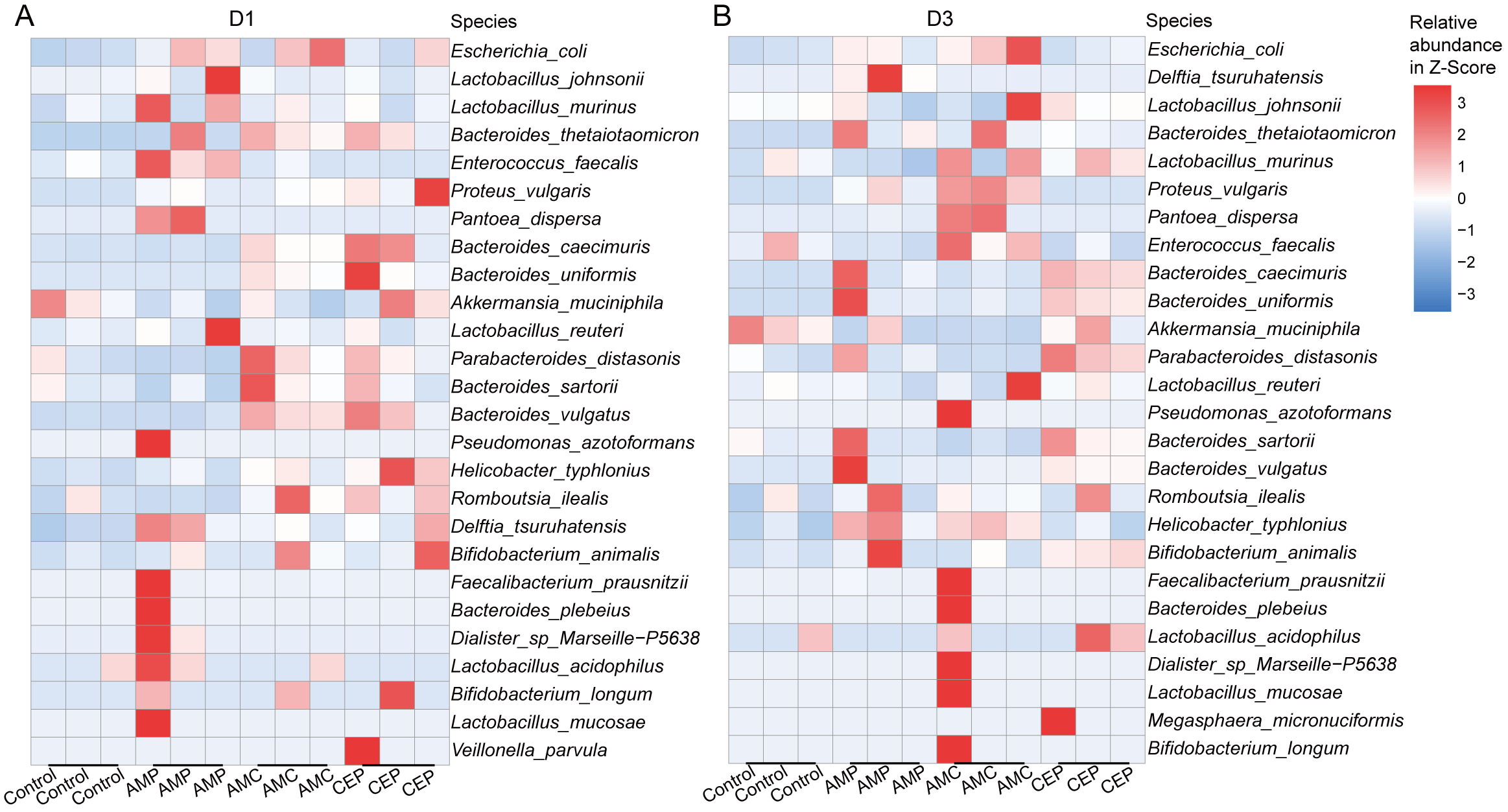

Supplement: Supplementary file 6 — Additional file 6. [file 12917_2025_4808_MOESM6_ESM.tif]
