# Supplementary material for: IncI2 plasmid transfer and changes of intestinal microbiota in mice under β-lactam antibiotic pressure
Source: BMC Vet Res. 2025 May 15;21:343. doi: 10.1186/s12917-025-04808-7 (PMC12080001; doi:10.1186/s12917-025-04808-7)
Supplement: Supplementary file 7 — Additional file 7. [file 12917_2025_4808_MOESM7_ESM.docx]

**Additional files**

**Additional file 1:** Source and species of recipient strains for transfer of IncI2 plasmid in the absence of β-lactam antibiotic selective pressure.

**Additional file 2:** MICs of donor and recipient strains used in this study.

**Additional file 3:** Concentration of antibiotics for different conjugative transfer system.

**Additional file 4:** Effects of different subinhibitory concentrations of β-lactam antibiotics on the growth of donor and recipient bacteria. (A-C) 1/2× MIC (D-F) 1/4× MIC and (G-I) 1/8× MIC.

**Additional file 5:** HGT of IncI2 plasmid in the mouse gut. Colonization levels in the mouse intestine for 96 h for (A) transconjugants and (B) recipient bacteria. (C) Conjugation transfer frequency of the IncI2 plasmid in the mouse intestine for 96 h.

**Additional file 6:** Heatmap of the relative abundance of microbial taxa at the species level following antibiotic treatment for (A) 1 day and (B) 3 days.
